# Photography and Medicine

**Published:** 1887-06

**Authors:** 


### EDITORIAL.

### PHOTOGRAPHY AND MEDICINE.

The remarkable advances made in many of the arts as a consequence of the adaptation of photography to their needs, has been fully equaled by the extent to which science—and especially medical science—has profited in the same direction. It is not alone in conjunction with the re-production of the field exhibited by the microscope that the value of photography has thus been demonstrated. Every department of medicine and surgery is now daily enriched by valuable concontributions made to the great storehouses of anatomy, histology, and pathology.

It is not only the teacher of medicine, but the student and the practitioner who are enjoying the fruit of this adaptation of one art to the requirements of another. What serves the one must, in the long run, greatly serve the other.

To-day all the facial expressions of disease of the internal organs are re-produced by the photograph; all the postures of hysteria; all the external manifestations of insanity. In surgery all the fractures, dislocations, and deformities are similarly reproduced. In one of the largest of the Philadelphia hospitals a camera has been arranged so as to secure accurate representations of persons suffering from shock, as the victims of accident are brought in on litters into the proper wards. In several of the medical schools the entire course in midwifery is illustrated by photographic representations. In diseases of the skin, though confronted with the almost *insur-*

*mountable* difficulty incident to the representation of colors, something has been done in the way of accurate illustration.

The object of calling attention to these facts, whose importance is rapidly increasing, respects the value to the physician of a practical acquaintance with the art of photography. There are few photographers limiting themselves to the popular demands upon their skill, who can satisfactorily accomplish what is required by the physician. The very best photographs of medical subjects have been taken by medical men. The degree of skill acquired by the latter in this particular field has been what we should expect of the man whose education is of the character to prepare him for such work. One of the busiest of the best-known physicians in this country was first to photograph successfully last year the flash of lightning. Some of the most artistic of the photographs of anatomical preparations have been also taken by physicians—not by those merely who have devoted themselves particularly to such work, but by practical men occupied daily with their professional duties.

The active practitioner, who has but little time for the recording of his interesting cases in writing will find here a field that will reward him for the small time required to cultivate it. No man has so excellent a record of the interesting features of a case of any disease that can be represented by a photograph, as that furnished by the camera. The modern methods of photography have been so simplified and improved, that the best artistic results are now within the reach of all.

